# Comparative effects of canagliflozin and sitagliptin in chronically ischemic myocardium

**DOI:** 10.20517/2574-1209.2023.95

**Published:** 2024-01-11

**Authors:** Sharif A. Sabe, Dwight D. Harris, Mark Broadwin, Cynthia M. Xu, Mohamed Sabra, Debolina Banerjee, M. Ruhul Abid, Frank W. Sellke

**Affiliations:** Division of Cardiothoracic Surgery, Department of Surgery, Cardiovascular Research Center, Rhode Island Hospital, Alpert Medical School of Brown University, Rhode Island Hospital, Providence, RI 02905, USA.

**Keywords:** Sodium-glucose cotransporter-2 inhibitor, dipeptidyl peptidase-4 inhibitor, coronary disease

## Abstract

**Aim:**

Recent studies demonstrate that sodium-glucose cotransporter 2 inhibitors (SGLT2i) and dipeptidyl peptidase-4 inhibitors (DPP4i), two classes of antidiabetic drugs, are cardioprotective. However, the mechanisms of these benefits and their comparative efficacy remain unclear. We aimed to compare the effects of these antidiabetic agents on cardiac function, perfusion, and microvascular density using a swine model of chronic myocardial ischemia.

**Methods:**

Chronic myocardial ischemia was induced in Yorkshire swine by ameroid constrictor placement to the left circumflex artery. Two weeks later, pigs were administered vehicle (“CON”, 8 pigs), 300 mg SGLT2i canagliflozin, (“CANA”, 8 pigs), or 100 mg DPP4i sitagliptin (“SIT”, 5 pigs) daily. Five weeks later, pigs were euthanized. Cardiac function, perfusion, collateralization, and protein expression were determined by pressure-volume catheter, microsphere analysis, immunofluorescence, and immunoblotting, respectively.

**Results:**

Compared with SIT, CANA was associated with improved stroke volume and cardiac output, with a trend towards reduced left ventricular stiffness. Both CANA and SIT trended towards improved perfusion compared to CON, but there were no differences between the two treatment groups. SIT was associated with improved capillary density with a trend towards improved arteriolar density compared to CANA. Both CANA and SIT were associated with increased expression of vascular endothelial cadherin compared to CON, without differences in treatment groups. SIT pigs had decreased 5′ adenosine monophosphate-activated protein kinase activation compared to CON and CANA. There was a trend towards increased endothelial nitric oxide synthase activation in the SIT group compared to CON. There were no differences in activation of extracellular signal-regulated kinase 1/2 across groups.

**Conclusions:**

In the setting of chronic myocardial ischemia, canagliflozin is associated with improved cardiac function compared to sitagliptin, with similar effects on perfusion despite differences in microvascular collateralization.

## INTRODUCTION

Longstanding coronary disease is a leading contributor to morbidity and mortality, and unfortunately, many patients with this disease process have poor revascularization options, leading to progressive tissue ischemia and subsequent myocardial adverse remodeling and heart failure^[1,2]^. Thus, it is important to identify cardioprotective medical therapies that reduce and/or reverse the deleterious effects of chronic coronary disease and ischemia.

There is a growing body of research and interest in antihyperglycemic drugs as cardioprotective agents independent of glucose control. Two antihyperglycemic drug classes that have been investigated include sodium-glucose cotransporter 2 inhibitors (SGLT2i) and dipeptidyl peptidase-4 inhibitors (DPP4i). SGLT2i have been shown in clinical trials to reduce the risk of cardiovascular death, stroke, and non-fatal myocardial infarction, irrespective of glucose control^[3–5]^. These findings prompted further exploration, which included small animal non-diabetic animal models of acute myocardial ischemia/infarction which showed that these agents reduce infarct size and improve cardiac function^[6–8]^. Building on these studies, our lab recently demonstrated that canagliflozin, an SGLT2i, improves cardiac function and perfusion in a non-diabetic swine study of ameroid constrictor-induced chronic myocardial ischemia (CMI)^[9]^. Likewise, another class of antihyperglycemic agents commonly used in clinical practice, DPP4i, may also have cardioprotective effects. Studies in murine models have shown that these agents improve infarct size and cardiac function^[10–12]^. Additionally, our lab recently demonstrated that sitagliptin, a DPP4i, improves cardiac perfusion via increased collateralization to chronically ischemic myocardium^[13]^.

While previous studies have demonstrated cardiovascular benefits of SGLT2i and DPP4i individually, it is important to understand the comparative effects of these agents given that they are typically clinically used for similar indications. Some clinical trials have attempted to address this area with varied results, highlighting the need for more direct mechanistic studies. For example, one clinical study showed that among patients with diabetes and heart failure, SGLT2i was associated with reduced risk of heart failure hospitalization, myocardial infarction, and stroke compared to DPP4 inhibitors^[14]^. Another trial comparing these agents in patients with diabetes and acute myocardial infarction found no differences in major adverse composite events, with improved left ventricular ejection fraction in patients receiving SGLT2i compared to DPP4i^[15]^. Finally, one trial compared the effects of empagliflozin, an SGLT2i, and sitagliptin on myocardial perfusion reserve in patients with diabetes and coronary disease, demonstrating similarly improved perfusion reserve across groups^[16]^. Unfortunately, direct functional and perfusion measurements are limited in these clinical studies, with unclear mechanisms of differential cardiovascular effects. Further, all of the aforementioned studies focused on patients with diabetes, though the comparative effects of these agents on non-diabetic subjects are still poorly investigated, an important consideration given that SGLT2i are increasingly prescribed to non-diabetic patients with cardiovascular disease^[17]^. These limitations in the current literature create major knowledge gaps in our understanding of the comparative effects of SGLT2i and DPP4i, which have yet to be addressed adequately.

In order to address these knowledge gaps, we sought to examine the comparative actions of SGLT2i and DPP4i in the context of longstanding coronary disease by utilization of our swine model of ameroid constrictor-induced myocardial ischemia. In this study, we compare the effects of SGLT2i canagliflozin and DPP4i sitagliptin on myocardial functional parameters, coronary perfusion, coronary microvascular density, and molecular signaling in a non-diabetic porcine model of CMI.

## METHODS

### Overview of study design

21 pigs (Yorkshire breed) that were 11 weeks old received a thoracotomy incision for placement of an ameroid constrictor to the left circumflex artery (LCxA). The ameroid constrictor produces CMI by absorption of heat and moisture and expansion inwardly to slowly occlude the artery, as reported by others^[18–20]^. Following a two-week period, the pigs were given either vehicle (CON, 8 pigs of which 3 were female and 5 were male), 300 mg oral canagliflozin daily (CANA, 8 pigs of which 4 were female and 4 were male), or 100 mg oral sitagliptin daily (SIT, 5 pigs of which 4 were male and 1 was female). Doses of medication were based on recommended doses for adult patients. Medications were administered orally by trained veterinary and research team staff. After five weeks of treatment, swine underwent euthanasia with myocardial tissue harvest for analysis. Per data from our prior experiments, we estimated the minimum sample size for significance using a 2 tailed α level of 0.01, ß error level of 0.10, and standard deviation for perfusion analysis of 0.150 mL/[mg·min], resulting in a minimum sample size of 7 per group. Of note, sitagliptin treatment was held for three weeks in two animals per the “RIH Institutional Animal Care and Use Committee” with early halting of this arm of the protocol due to mortality as described previously resulting in a smaller sample size in the SIT group^[13]^. This study was approved by the RIH IACUC (#505821).

### Ameroid constrictor placement

An ameroid constrictor was placed on the proximal LCxA of all pigs in the study. The pigs received oral 30 mg/kg cephalexin and 10 mg/kg aspirin one day before and five days after the surgery. A fentanyl patch of 4 ug/kg was applied just before the surgery and kept for 72 h. The pigs were given an intramuscular injection of 4.4 mg/kg telazol and 2.2 mg/kg xylazine to induce anesthesia. The pigs underwent intubation and mechanical ventilation, while isoflurane inhalation was employed for anesthesia maintenance. Intravenous administration of normal saline at a rate of 5 mL/kg/h was initiated. Supine positioning of the pigs followed, with sterile preparation and draping preceding a mini-thoracotomy on the left side. Subsequently, sharp entry was made into the pericardium for visualization of the left atrium, allowing for retraction and visualization of the LCxA close to its branching off from the left main coronary artery. To temporarily obstruct blood flow through the LCxA, a loop was placed around it after systemic heparinization. The LCxA was effectively blocked for a duration of 22 min by elevating the loop, verified by observable changes in ST and/or T waves on the electrocardiogram (ECG). This period of LCxA occlusion coincided with the injection of 5 mL of gold-labeled microspheres into the left atrial cavity. Upon releasing the vessel loop and restoring LCxA blood flow, ECG alterations were monitored until they reverted to normal. A hygroscopic casein ameroid constrictor containing a titanium case was placed circumferentially around the LCxA. The surgical incision was sutured in multiple layers for proper reapproximation.

### Terminal harvest

The terminal harvest procedure was performed five weeks after treatment. The pigs received systemic heparinization (80 IU/kg). Midline sternotomy was performed. Resting and pacing blood flow analyses at 150 bpm were done by injecting 5 mL of isotope-labeled microspheres into the left atrium while simultaneously withdrawing 10 mL of blood from the femoral artery catheter. Hemodynamic measurements were obtained by placing a pressure-volume (PV) catheter via a 6F sheath to the left ventricular apex. The heart was removed at the end of the procedure, and heart tissue was quickly separated into 16 different segments corresponding to the distribution of the left cirumflex artery and left anterior descending artery. Myocardial tissue segments were either dried in a warm oven and then stored for microsphere-based studies or submerged in liquid nitrogen and then frozen at −80 °C for western blot experiments and frozen sections.

### Myocardial perfusion measurements

Using isotope-labeled microspheres given during ameroid and harvest procedures, myocardial perfusion was assessed. To define the left ventricle’s perfusion territory by the LCxA, 5 mL of gold-labeled microspheres were injected into the left atrial appendage while employing a vessel loop to occlude LCxA. During the harvesting procedure, 5 mL of Lutetium-labeled microspheres were introduced into the left atrium, and concurrently, 10 mL of blood was withdrawn from the femoral artery at a fixed rate via a withdrawal pump. Samarium-labeled microspheres were used for the same protocol during pacing at 150 bpm. Samples of blood and myocardial tissue from 10 distinct sections were collected, and selected based on their proximity to both the LAD and LCxA arteries. The weight of these samples was obtained, and then they were subjected to oven-drying, and subsequently forwarded to the Biophysics Assay Laboratory for analysis of microsphere density and blood flow calculations.

### Cardiac functional measurements

To collect cardiac functional assessments while conducting the harvest procedure, a PV catheter was directly introduced into the left ventricle’s apex. Throughout breath holds, load-dependent information was gathered to mitigate the influence of respiratory fluctuations, while during respiratory holds and vessel loop occlusion of the IVC using a vessel loop, load-independent data were acquired. Hemodynamic parameters were documented and processed using “LabChart” software. Measurements collected included stroke volume (SV), stroke work (SW), cardiac output (CO), left ventricular stiffness (ß), preload recruitable stroke work (PRSW), and dP/dt max.

### Microvessel quantification

Immunofluorescence staining was used to determine microvessel density, as described previously^[21]^. Primary and secondary antibodies used in this protocol are listed in Supplementary Table 1. Images were examined at 20× magnification using an Olympus VS200 Slide Scanner. Image analysis was conducted using QuPath software^[22]^. Capillary density was assessed by determining the percentage of tissue area stained through the thresholding of positive isolectin B4 staining. Arteriolar density was analyzed by defining positive SMA staining through thresholding and calculating the object number per tissue section area.

### Immunoblotting studies

Immunoblotting studies were performed as previously described^[9]^. Primary and secondary antibodies used in this protocol are listed in Supplementary Table 1. Material sources are listed in Supplementary Table 2. NIH Image J software was used for band density densitometry.

### Data analysis

Median values with interquartile ranges are used to present all data. The statistical analysis was performed using the Wilcoxon rank-sum test, and Holm correction was applied for multiple comparisons, utilizing R software. Probability values less than 0.05 were deemed statistically significant.

## RESULTS

### Canagliflozin improves cardiac systolic and diastolic functional parameters compared to sitagliptin

**Figure 1.**
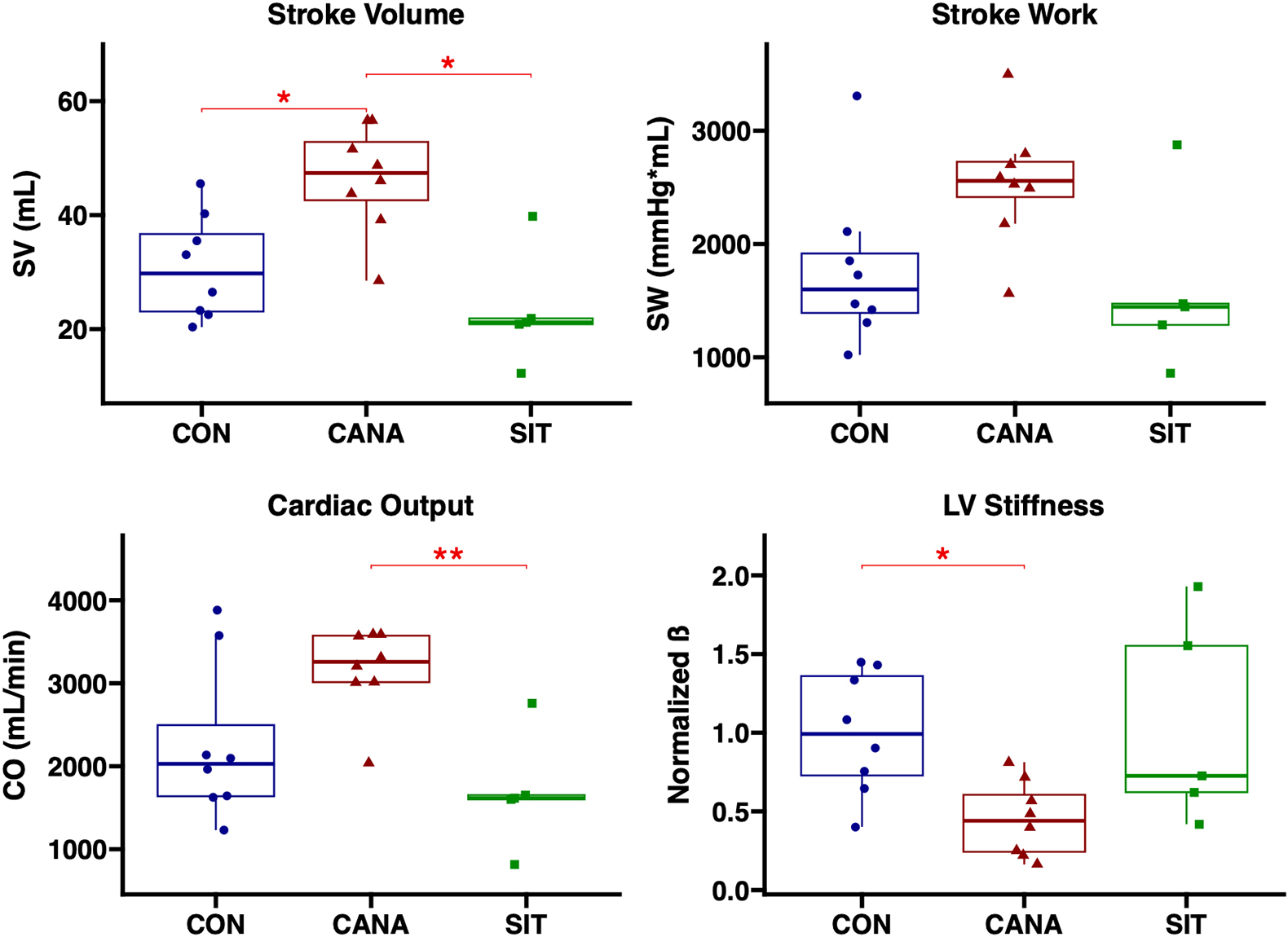
Canagliflozin improves cardiac systolic and diastolic functional parameters compared to sitagliptin in swine with chronic myocardial ischemia. Cardiac functional measurements of stroke volume (SV), stroke work (SW), cardiac output (CO), and coefficient of LV stiffness are shown in swine treated with canagliflozin (CANA, *n* = 8), sitagliptin (SIT, *n* = 5), or vehicle with no drug (CON, *n* = 8). *signifies a *P* value of less than 0.05. **signifies a *P* value of less than 0.01.

Canagliflozin was associated with increased SV compared to control, with trends towards improved SW (*P* = 0.062) and CO (*P* = 0.21), while sitagliptin was associated with a trend towards decreased SV (*P* = 0.13) and CO (*P* = 0.22) compared to control. Compared to CANA-treated swine, SIT-treated swine had decreased SV (*P* = 0.019) and CO (*P* = 0.009). Canagliflozin was associated with decreased LV stiffness (ß) compared to control (*P* = 0.021), with a trend towards decreased stiffness compared to sitagliptin-treated swine (*P* = 0.13). No significant differences were noted in contractility as measured by dP/dt max (*P* > 0.5 for all comparisons) by the slope of the PRSW equation (*P* > 0.5 for all comparisons) across groups [Figure 1].

### Canagliflozin and sitagliptin are associated with comparable improvements in perfusion to chronically ischemic myocardium

**Figure 2.**
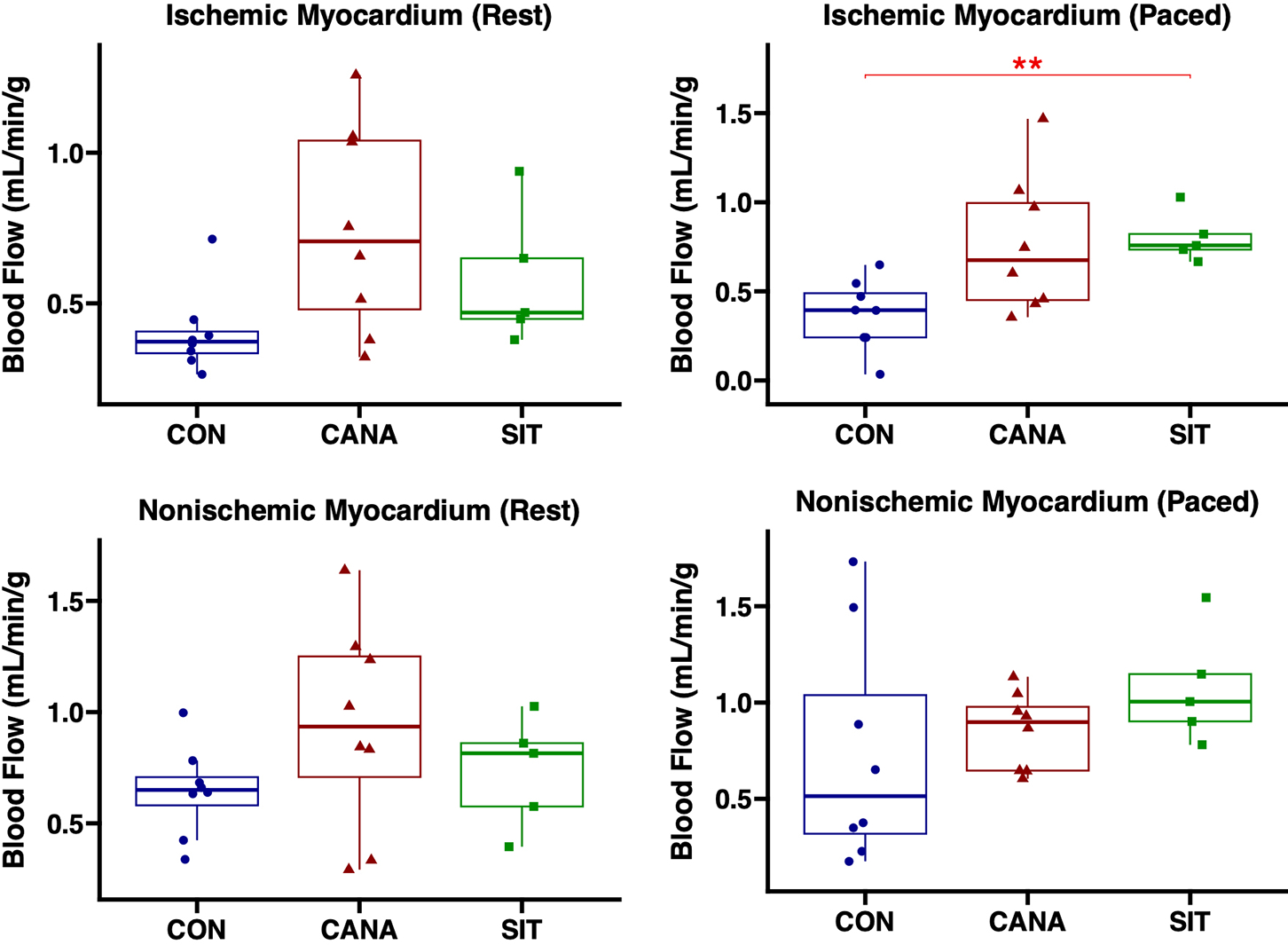
Canagliflozin and sitagliptin therapy demonstrate comparable improvements in coronary perfusion to chronically ischemic myocardium. Perfusion expressed as mL/min/g as measured by microsphere analysis shown at rest and during pacing to 150 beats/min in pigs receiving canagliflozin (CANA, eight animals), sitagliptin (SIT, five animals), or vehicle with no drug (CON, eight animals). ^★★^signifies a *P* value of less than 0.01.

Both canagliflozin and sitagliptin groups demonstrated increased perfusion in ischemic myocardium compared to control at rest (CAN *P* = 0.11, SIT *P* = 0.11) and at pacing conditions to 150bpm (CAN *P* = 0.08, SIT *P* = 0.005), without significant differences between treatment groups (*P* = 0.44 at rest, *P* = 0.62 at pacing). In nonischemic myocardium, no changes in coronary perfusion were noted compared to control at rest (CAN *P* = 0.48, SIT *P* = 0.87) or during pacing (CAN *P* = 0.51, SIT *P* = 0.513). No changes in coronary perfusion were noted in nonischemic territory between treatment groups (*P* > 0.5 at rest and during pacing) [Figure 2].

### Sitagliptin is associated with improved coronary microvessel density compared to canagliflozin

**Figure 3.**
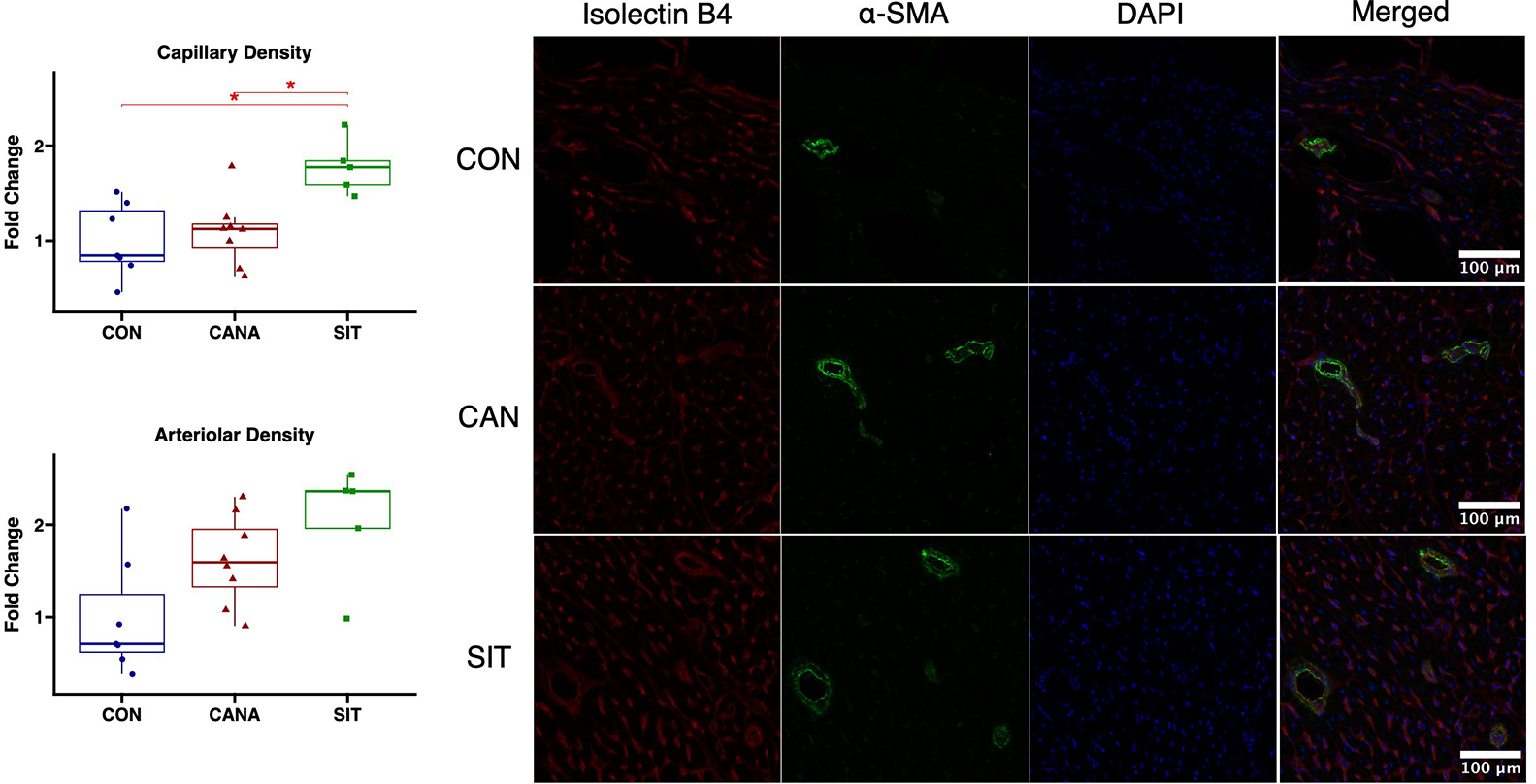
Sitagliptin increases coronary microvessel collateralization in chronically ischemic myocardium compared to canagliflozin. Capillary and arteriolar density in swine treated with canagliflozin (CANA, eight animals), sitagliptin (SIT, five animals), and vehicle containing no medication (CON, seven animals) shown on box and whisker plots.

Sitagliptin-treated swine had improved arteriolar density (*P* = 0.05) and capillary density (*P* = 0.015) in chronically ischemic myocardium compared to control. Compared to canagliflozin-treated swine, sitagliptin-treated swine had improved capillary density (*P* = 0.022) with a trend towards improved arteriolar density (*P* = 0.14). (Figure 3, complete blots are shown in Supplementary Figure 1).

### Sitagliptin treatment is associated with reduced AMPK activation compared to canagliflozin in chronically ischemic myocardium

**Figure 4.**
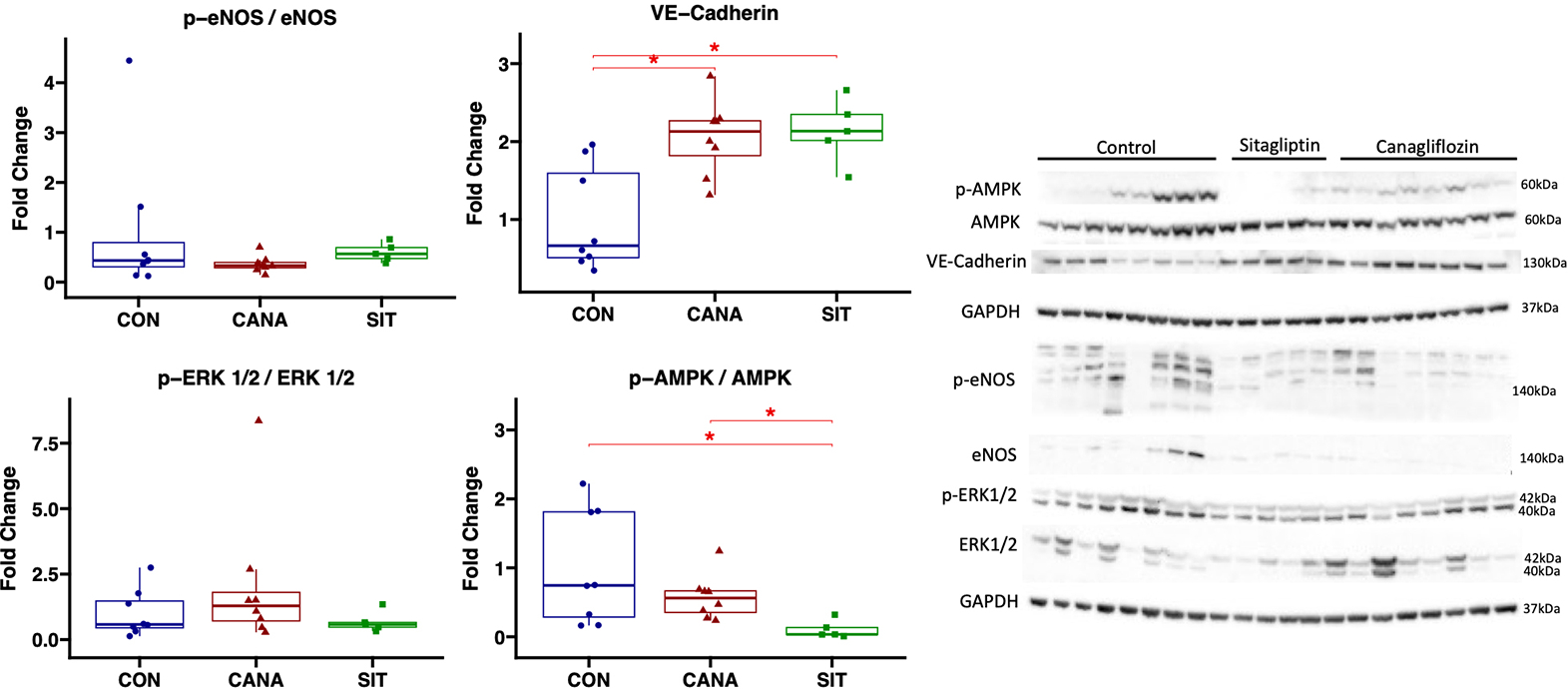
Immunoblot results in chronically ischemic myocardium with canagliflozin and sitagliptin. Data shown from immunoblot experiments in chronically ischemic myocardium in swine treated with canagliflozin (CANA, *n* = 8), sitagliptin (SIT, *n* = 5), and vehicle with no drug (CON, *n* = 8). ^★^signifies a *P* value of less than 0.05.

**Figure 5.**
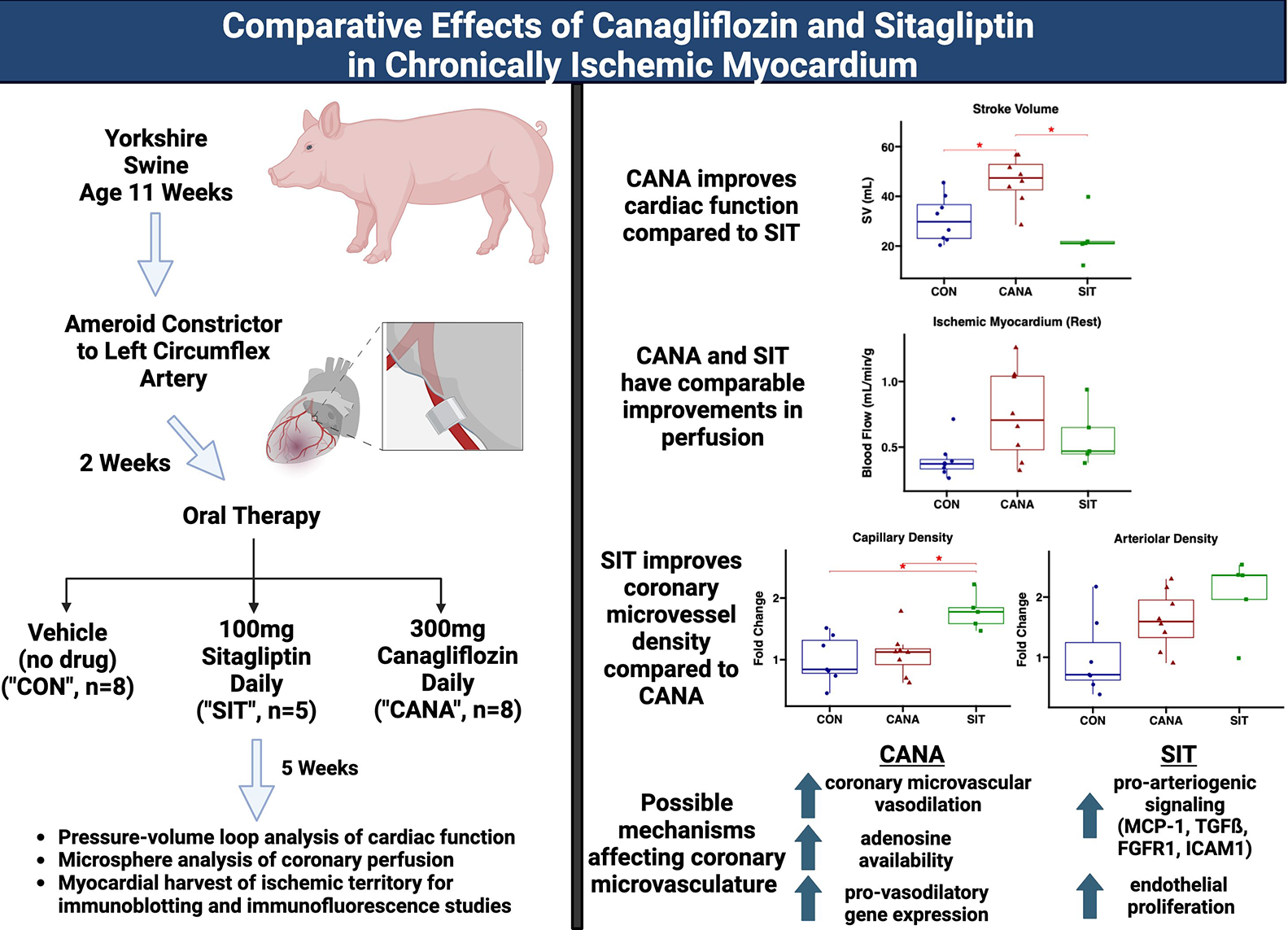
Graphical abstract. All Yorkshire swine age 11 weeks had an ameroid constrictor placed to the left circumflex artery. Two weeks later oral therapy was started with vehicle (“CON”, *n* = 8), sitagliptin (“SIT”, *n* = 5), or canagliflozin (“CANA”, *n* = 8) daily. Five weeks after treatment cardiac functional analysis was performed and tissue was harvested for further analysis. Main findings included improved cardiac function with CANA compared to SIT, similar improvements in perfusion with the two treatment agents, and improved microvascular density with SIT. Possible mechanisms implicated in coronary perfusion and collateralization are also outlined. CON: Control; SIT: sitagliptin; CANA: canagliflozin; MCP-1: monocyte chemoattractant protein 1; TGFß: tumor growth factor beta; FGFR1: fibroblast growth factor receptor 1; ICAM 1: intercellular adhesion molecule 1.

In the ischemic myocardium of the canagliflozin-treated pigs, immunoblot experiments showed decreased activation of AMPK in SIT-treated pigs compared to CON pigs (*P* = 0.019) and CANA-treated swine (*P* = 0.019) as determined by the p-AMPK to AMPK ratio. Compared to canagliflozin-treated swine, sitagliptin trended towards increased activation of eNOS (*P* = 0.14), without difference in activation of ERK1/2 (*P* = 0.67). Both canagliflozin and sitagliptin were associated with increased expression of VE-Cadherin compared to control (*P* = 0.014 for both comparisons), without differences between treatment groups (*P* = 0.62). [Figures 4 and 5].

## DISCUSSION

In the present study, we utilized a large animal model of CMI to compare the cardiac effects of canagliflozin, a SGLT2i, and sitagliptin, a DPP4i. We determined that in the setting of CMI, CANA improved cardiac function compared to SIT, with similar effects on perfusion despite improved coronary microvessel collateralization in SIT-treated pigs. Sitagliptin was associated with reduced AMPK activation compared to canagliflozin in chronically ischemic myocardium. These findings together highlight the differing cardiac effects of two important classes of antihyperglycemic agents in the absence of diabetes.

SGLT2i have shown significant promise as a cardioprotective agent in basic and clinical studies. They have been shown to reduce the risk of cardiovascular death, stroke, and non-fatal myocardial infarction, irrespective of the presence of diabetes^[3–5,23–28]^. These positive trials prompted the recommendation of these agents in patients with heart failure even in the absence of diabetes^[17]^. Animal studies investigating mechanistic explanations for these benefits have shown that SGLT2i reduce infarct size, improve cardiac function, attenuate oxidative stress, and improve endothelium-dependent vasodilation^[7–9]^. Another commonly used class of antihyperglycemic agents, DPP4i, have shown cardioprotective effects in small and large animal models, including improved cardiac function, reduced apoptosis and oxidative stress, attenuated fibrosis, and improved endothelial proliferation^[10–13]^. We have previously investigated the effects of canagliflozin and sitagliptin on cardiac function and microvascular collateralization and perfusion in our large animal model of chronic myocardial ischemia, and we previously discussed these effects in relation to prior studies. However, to our knowledge, the present study is the first to investigate the comparative effects of these agents on cardiac function and coronary microvasculature using a large animal model of chronic myocardial ischemia.

Previous clinical trials have attempted to address the differential effects of SGLTi and DPP4i on cardiovascular-related outcomes. Gonzalez and others retrospectively analyzed Medicare data to investigate the differential effects of DPP4i, SGLT2i, and another class of antihyperglycemic agents, glucagon-like peptide-1 receptor agonists, on clinical outcomes in patients with diabetes and heart failure with reduced and preserved ejection fraction^[14]^. They found that SGLT2i was associated with a reduced risk of heart failure hospitalization compared to the other agents, with a reduced risk of MI and stroke compared to DPP4i^[14]^. This study, unfortunately, lacks clarity on the differential effects on myocardial function, an important endpoint in evaluation of the effects of medical therapies on chronic coronary disease, given that untreated myocardial ischemia leads to functional deterioration and heart failure. Others have attempted to bridge this gap, with one retrospective study demonstrating improved left ventricular ejection fraction with SGLT2i compared to DPP4i in patients with diabetes and cardiovascular disease^[29]^, and another study demonstrating concurrent SGLT2i use with metformin resulting in improved left ventricular ejection fraction compared to concurrent DPP4i use with metformin^[15]^. However, studies are lacking on the differential effects of these agents in the absence of diabetes, an important consideration given the increased use of these agents in non-diabetic patients given cardioprotective effects. The current study provides some clarity in this regard with direct functional measurements using a PV catheter inserted into LV cavity. Our findings highlight that even in non-diabetic animals, canagliflozin augments cardiac functional parameters compared to sitagliptin therapy; the latter, in fact, may result in somewhat diminished cardiac systolic function compared to control.

We also investigated the differential effects of canagliflozin and sitagliptin on microvascular collateralization and perfusion in chronically ischemic myocardium. We found that sitagliptin, compared to canagliflozin therapy, improved capillary and arteriolar collateralization. Interestingly, despite these differences in collateralization, no notable changes were noted in myocardial coronary perfusion within ischemic myocardial regions between treatment groups. These findings are consistent with findings from a randomized clinical trial by Oh *et al.*, who investigated the effects of empagliflozin, an SGLT2i, with sitagliptin on myocardial perfusion reserve in patients with coronary disease and diabetes using single-photon emission computed tomography imaging^[16]^. Their study demonstrated improvement in myocardial perfusion reserve with both agents, but no significant difference between treatment groups^[16]^. Similar to the limitations of comparative studies of SGLT2i and DPP4i with regard to cardiac function, there is a lack of studies exploring the impact of these medications in the absence of diabetes. Within the current study, our direct perfusion measurements using isotope-labeled microspheres demonstrated improved perfusion with both sitagliptin and canagliflozin therapy without notable differences between groups. We have previously demonstrated improved perfusion with canagliflozin therapy despite a lack of microvessel collateralization^[9]^, which may be secondary to improvements in vasodilatory function in the coronary microvasculature with these agents. We have recently demonstrated that canagliflozin improves coronary microvascular vasodilation and decreases vasoconstriction independent of angiogenesis, possibly via increased pro-vasodilatory metabolite availability and gene expression^[30]^. These findings may explain why canagliflozin has similar improvements to perfusion as collateral-dependent improvements in perfusion seen with sitagliptin therapy [Figure 5].

We utilized immunoblotting experiments to investigate some preliminary molecular pathways in order to determine how SGLT2i and DPP4i differentially affect chronically ischemic myocardium. Interestingly, though there was a trend towards increased activation of angiogenic marker eNOS in sitagliptin-treated compared to canagliflozin-treated swine, there were no differences in activation of angiogenic marker ERK1/2, or in expression of VE-cadherin between treatment groups. Therefore, there are likely alternative mechanisms involved in the differential collateralization response between these two agents. One important finding was the decreased activation of AMPK in the sitagliptin group compared to the canagliflozin group. AMPK plays a major role in a variety of cardiovascular functions, including metabolism, transcription, mitochondrial function, and contractility^[31,32]^. Decreased AMPK activation may, in part, contribute to reduced cardiac function with sitagliptin therapy compared to canagliflozin, which may offset the beneficial effects on perfusion.

In this study, we focused on the effects of SGLT2i and DPP4i on myocardial function, the coronary microvasculature, and related molecular mechanisms. However, there are likely other mechanisms involved in the cardioprotective effects of these agents that have been demonstrated previously, including effects on inflammation, fibrosis, and metabolism^[9,13,33]^. All of these effects are likely independent of glucose control, given that these experiments were performed in animals receiving a normal diet. We have reported some of these effects in SGLT2i and DPP4i independently^[9,13,33]^, but further investigative studies comparing the effects of these agents on those and other pathways may provide further insight into the differential effects of SGLT2i and DPP4i in chronically ischemic myocardium.

### Study limitations

There are important limitations to consider in this study. One important limitation is the small sample size in our large animal model, which may have underpowered our study, particularly with statistical correction for multiple comparisons. Another limitation is in the single-time point of analysis, five weeks after therapeutic agent initiation, which limits our understanding of the differential effects of these agents with short- and longer-term use and may underestimate the dynamic changes that occur with time. Additionally, to increase clinical relevance, we dosed medications based on clinically used doses, given that the weight of pigs at the time of harvest is similar to that of a normal adult patient. However, at the time medications are initially administered, the pigs are smaller, and doses of medications at that time may be physiologically higher per weight than clinically relevant, which may exaggerate the molecular and functional effects of the medications. Additionally, our small animal model utilizes adolescent Yorkshire swine, given practical and logistical limitations to using older and larger swine; however, studies into the effects of these antihyperglycemic agents in animal models of aging, with its associated endothelial dysfunction and neovascularization^[34]^, may provide valuable insights particularly given some promising trials in the setting of frailty^[35,36]^. Finally, there are several agents within the classes of SGLT2i and DPP4i, and we only investigated one within each class. However, the mechanistic effects of these agents within each class may vary, and the comparison of additional agents may yield interesting results.

In summary, in the setting of CI, canagliflozin is associated with improved cardiac function compared to sitagliptin, with similar effects on perfusion despite differences in microvascular collateralization. These findings indicate that despite differing specific cardiovascular effects, canagliflozin therapy may result in an overall greater benefit in the context of CMI compared to sitagliptin treatment, though further studies on the comparative effects of these agents may be warranted.

We would like to thank the veterinary and animal care staff at Rhode Island Hospital for their excellent care of the animals used in this study.

## Supplementary Material

supplementary material

## Data Availability

The data that support the findings of this study are available from the corresponding author upon reasonable request.
