# Supplementary material for: Comparative effects of canagliflozin and sitagliptin in chronically ischemic myocardium
Source: Vessel Plus. Author manuscript; Available in PMC 2024 Aug 22. (PMC11339913; doi:10.20517/2574-1209.2023.95)
Supplement: supplementary material [file NIHMS1965311-supplement-supplementary_material.pdf]

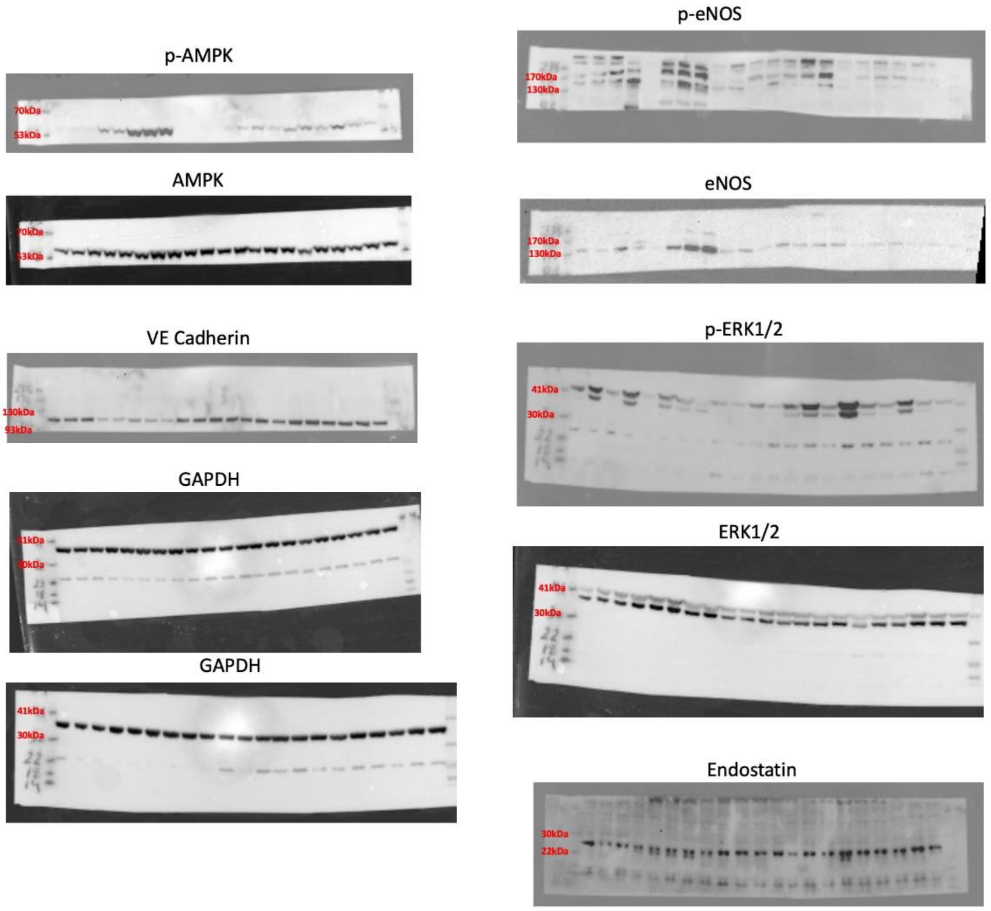

Supplemental Figure 1: Complete immunoblots. p-, phosphorylated; AMPK 5' adenosine monophosphate-activated protein kinase (AMPK); p-, phosphorylated; ERK1/2, extracellular signal-regulated kinase 1/2; eNOS, endothelial nitric oxide synthase; VE-Cadherin, vascular endothelial cadherin; GAPDH, glyceraldehyde 3-phosphate dehydrogenase.

Supplemental Table 1: Antibody Catalog Numbers

**Antibody Name**	**Manufacturer**	**Catalog Number**	**Concentration**
AMPK	Cell Signaling	2532	1:1000
Anti-rabbit IgG, HRP-linked Antibody	Cell Signaling	7074	1:4000
Anti-mouse IgG, HRP-linked Antibody	Cell Signaling	7076	1:4000
eNOS	Cell Signaling	32027	1:1000
ERK1/2	Cell Signaling	4695	1:2000
GAPDH	Cell Signaling	97166	1:1000
Isolectin B4	Thermo Fisher Scientific	I32450	1:100
p-AMPK	Cell Signaling	2535	1:1000
p-eNOS	Cell Signaling	9571	1:1000
p-ERK1/2	Cell Signaling	4370	1:1000
VE-Cadherin	Cell Signaling	2500	1:1000
α-SMA	Abcam	7817	1:500

Antibodies used in this study are listed along with corresponding manufacturer and catalog numbers. AMPK, 5’ adenosine monophosphate-activated protein kinase; IgG, immunoglobulin G; HRP, horseradish peroxidase; eNOS, endothelial nitric oxide synthase; ERK, extracellular regulated kinase 1/2; GAPDH, glyceraldehyde-3-phosphate dehydrogenase; VE-cadherin, vascular endothelial cadherin; α-SMA, alpha smooth muscle actin; p-, phosphorylated.

Supplemental Table 2: Material/Equipment Sources

**Material/Equipment**	**Manufacturer**	**Location**
Yorkshire Swine	Tufts	Boston, MA
Ameroid Constrictor	Research Instruments SW	Escondido, MA
Canagliflozin	Janssen Pharmaceuticals	Beerse, Belgium
Sitagliptin	Merck & Co.	Rahway, NJ
Pressure-Volume Catheter	Transonic	Ithica, NY
Microspheres	Biophysics Assay Laboratory	Worcester, MA
Withdrawal Pump	Harvard Apparatus	Holliston, MA
LabChart Software	ADInstruments	Colorado Springs, CO
Olympus VS200 Slide Scanner	Olympus Corporation	Tokyo, Japan
Radioimmunopreciptation Asssay Buffer	Boston Bioproducts	Milford, MA
Bis-Tris Gel	Thermo Fisher Scientific	Waltham, MA
Digital Camera System	Bio-Rad ChemiDoc MP, Life Science	Hercules, CA
Chemiluminesence ECL Solution	Thermo Fisher Scientific	Waltham, MA
